# Expanding the horizons of microRNA bioinformatics

**DOI:** 10.1261/rna.065565.118

**Published:** 2018-08

**Authors:** Rachael P. Huntley, Barbara Kramarz, Tony Sawford, Zara Umrao, Anastasia Kalea, Vanessa Acquaah, Maria J. Martin, Manuel Mayr, Ruth C. Lovering

**Affiliations:** 1 Institute of Cardiovascular Science, University College London, London WC1E 6JF, United Kingdom; 2 European Bioinformatics Institute, European Molecular Biology Laboratory (EMBL-EBI), Wellcome Trust Genome Campus, Cambridge CB10 1SD, United Kingdom; 3 King's British Heart Foundation Centre, King's College London, London SE5 9NU, United Kingdom

**Keywords:** microRNA, Gene Ontology, functional annotation, bioinformatic resource, network analysis, pathway analysis

## Abstract

MicroRNA regulation of key biological and developmental pathways is a rapidly expanding area of research, accompanied by vast amounts of experimental data. This data, however, is not widely available in bioinformatic resources, making it difficult for researchers to find and analyze microRNA-related experimental data and define further research projects. We are addressing this problem by providing two new bioinformatics data sets that contain experimentally verified functional information for mammalian microRNAs involved in cardiovascular-relevant, and other, processes. To date, our resource provides over 4400 Gene Ontology annotations associated with over 500 microRNAs from human, mouse, and rat and over 2400 experimentally validated microRNA:target interactions. We illustrate how this resource can be used to create microRNA-focused interaction networks with a biological context using the known biological role of microRNAs and the mRNAs they regulate, enabling discovery of associations between gene products, biological pathways and, ultimately, diseases. This data will be crucial in advancing the field of microRNA bioinformatics and will establish consistent data sets for reproducible functional analysis of microRNAs across all biological research areas.

## INTRODUCTION

To understand the basis of any given disease it is necessary to know the functions of the genes involved and the pathways they act in. There is a vast amount of functional knowledge available but it is a formidable task for researchers to become familiar with the roles of every gene in their particular disease or trait of interest. Thus, bioinformatic resources are widely used to navigate this knowledge in a fast and efficient way. Many resources available are focused on protein-coding genes, but with the discovery that microRNAs (miRNAs) are important regulatory molecules, it is essential that the bioinformatic resource space be expanded to offer the same wealth of reliable functional information for miRNA research.

MicroRNAs are small, ∼22-nucleotide molecules that act by binding to complementary target mRNA strands in order to silence their gene expression (Filipowicz et al. 2008). Research into miRNAs is a relatively new and fast growing field generating a considerable volume of experimental data. A whole plethora of miRNA bioinformatic databases and tools have been developed that include information on expression, sequence, and target data (Akhtar et al. 2016), however there are few that offer high-quality information on the direct functions of miRNAs and their roles in the regulation of biological pathways. One tool that does provide functional data, FAME (Ulitsky et al. 2010), does not provide direct functional information, but infers the cellular role of individual miRNAs based on the Gene Ontology (GO) and KEGG annotations associated with the computationally predicted target genes of each miRNA. Another resource, the Human microRNA Disease Database (HMDD), curates miRNA-disease associations from the literature and this data can be used to infer miRNA function based on the miRNA disease-phenotype grouping (Li et al. 2014). Researchers wishing to perform functional analysis of miRNA function, however, largely have no choice but to perform indirect analysis via the roles of the predicted targets using functional analysis tools developed for analyzing protein-coding genes (Tang et al. 2016; Yan et al. 2016; Yuan et al. 2016). This is problematic because the varied scoring and prediction techniques that are currently in use by prediction tools to identify target mRNAs mean that there is very little agreement on which mRNAs are targeted by any given miRNA (Jovanovic et al. 2010; Kast 2011). In addition, the majority of tools or algorithms predict that most miRNAs will silence hundreds to thousands of genes (Jovanovic et al. 2010; Kast 2011; Singh 2017). Akhtar and coworkers found that the most common limitation of miRNA target prediction tools is their generation of large amounts of false positive data (Akhtar et al. 2016). Consequently, many of the predicted target genes may not be physiological targets of the miRNAs and yet their roles in multiple, and often diverse, pathways are being included in analyses of miRNA function, thus leading to errors in predicting the biological role of individual, or groups of, miRNAs. The problems associated with this type of analysis is illustrated by a reduction in significance values of the enriched biological processes associated with miRNAs, when target distribution bias is corrected (Bleazard et al. 2015). Tools are being developed to address this bias by incorporating miRNA-specific data, including tissue expression, family, function, target, and disease association, from a variety of sources in order to perform enrichment analysis. Two such tools are TAM and miEAA, and since these tools are dependent on integrated data, as the quality and volume of data improves, so will the quality of the analyses (Lu et al. 2010; Backes et al. 2016). Additionally, some curated miRNA target databases are trying to reduce false-positives by providing experimentally verified target information for individual miRNAs (Xiao et al. 2009; Chou et al. 2015; Dweep and Gretz 2015; Karagkouni et al. 2017). Although some of these miRNA databases are evidently well managed and curated, there has long been concern over the quality of data in others. An investigation into the accuracy and completeness of four databases that claim to contain validated miRNA:target interactions was undertaken by the Witwer group (Lee et al. 2015b). The study found that the databases varied widely in their results for the same query, were not consistent over time and the supporting evidence for miRNA:target interactions was largely indirect or weak, concluding that these databases should be used with caution and the miRNA targets substantiated by checking the primary literature.

The problem, thus, is twofold: (i) the inclusion in analyses of predicted, potentially erroneous, miRNA functional information, which is based on the functional annotations associated with false-positive miRNA targets and (ii) a deficit of miRNA annotations that describe the known role of miRNAs in individual biological pathways that can be used directly for miRNA functional analysis. This combination has severe consequences on data analysis, data interpretation and hypothesis testing.

Here we present two new data sets: the first consisting of GO annotations describing the experimentally proven biological roles of miRNAs, which can be used in analyses to identify processes regulated by a specific miRNA or a set of miRNAs; the second consisting of experimentally validated miRNA:target interactions, which will enable confident identification of validated miRNA targets, as well as indirect analysis of miRNA function based on the established role of mRNAs proven to be targeted by the miRNA(s).

The GO is widely regarded as an indispensable resource that has proven to be especially useful to researchers in guiding their research aims (Ashburner et al. 2000). The GO annotation data set is easily accessible by both researchers investigating small data sets and bioinformaticians performing complex computational analyses (Balakrishnan et al. 2013; The Gene Ontology Consortium 2017). Additionally, the major biological databases, such as Ensembl (Zerbino et al. 2017), UniProtKB (UniProt Consortium 2017), NCBI Gene (NCBI Resource Coordinators 2017), GeneCards (Stelzer et al. 2016), and even Wikipedia, display the GO annotations available for each gene or gene product. The GO Consortium (The Gene Ontology Consortium 2017) has largely focused on biocuration of protein-coding genes, primarily due to the scarcity, until recent years, of experimental data for noncoding genes, including miRNAs. Our group at University College London (http://www.ucl.ac.uk/functional-gene-annotation), as a member of the GO Consortium, has extended the GO data set to include manually curated functional annotations for mammalian miRNAs, thereby creating a novel data set for use in miRNA research. These annotations are freely available via specific GO browsers (such as QuickGO [Binns et al. 2009] and AmiGO [The Gene Ontology Consortium 2017]), as well as from miRBase (Kozomara and Griffiths-Jones 2014), Ensembl, NCBI Gene, and in file downloads from EMBL-EBI (Cook et al. 2017). In addition, using our experimentally verified miRNA:target interaction GO annotations, we have created a new data set that is accessible from the PSICQUIC web service (del-Toro et al. 2013) and from within network analysis tools, such as Cytoscape (Shannon et al. 2003).

Manual curation using GO involves expert biocurators reading primary experimental literature to gather biological information and then representing this information as GO annotations (Balakrishnan et al. 2013). The information captured for miRNAs in the new GO data set comprises both their functional roles, i.e., the processes they regulate or are part of, and their experimentally validated target genes, supplemented with physiologically relevant contextual information where there is sufficient experimental evidence (Huntley et al. 2014). The functional aspect of the GO annotations will be vital for direct analysis of miRNA function, and when combined with the validated miRNA target data that we provide and the GO annotations associated with the targets of the miRNA (provided by the GO Consortium) will strengthen the confidence of the analysis. The contextual information we provide in the GO annotations, including the cellular and/or anatomical location of the miRNAs’ function, will greatly enhance pathway and network analyses, where it is important to distinguish the cell or tissue types in which certain pathways occur or certain miRNA:target pairs act (Khatri et al. 2012).

This article describes the need for manual functional annotation of miRNAs using, as examples, the provenance of miRNA:target interactions from two popular curated miRNA target databases, thus illustrating how miRNA research is currently hampered by unreliable or missing data. With this we hope to educate users in the problems that they need to consider when investigating the specific biological roles of miRNAs. To begin to address these problems, we introduce the miRNA GO annotation and miRNA:target interaction data sets, which provide a novel and high-quality manually curated resource for miRNA research. As sole providers, currently, of human miRNA GO annotations, we prioritize curation of experimental information for human miRNAs. Occasionally, however, experimental information is lacking for a given human miRNA. In these cases mammalian orthologs may be curated and, using strict criteria that we describe, transferred to the human miRNA. We go on to illustrate how our data can be used to create miRNA-focused interaction networks with a biological context, thereby enhancing miRNA-related data analysis. As our miRNA annotations are now available in several high-profile biological databases, we anticipate they will begin to be incorporated into popular functional analysis tools, thereby improving interpretation of miRNA experimental data.

## RESULTS

### Investigating data quality in two miRNA target databases

In order to confirm the necessity for a manually curated, experimentally verified functional information resource for miRNAs, an assessment of data quality in two miRNA target databases was performed. Databases such as these aim to supply information about miRNA targets in volumes large enough to be useful for bioinformatic analysis, therefore it is understood that some errors are unavoidable. It is useful, however, to assess the quality of data provided for an individual miRNA in order to measure the possible rate of error.

Two of the most highly cited miRNA target databases were chosen, miRTarBase (Chou et al. 2015), which appears in 70 articles in PubMed (up to November 21, 2017) and miRWalk (Dweep and Gretz 2015), which appears in 104 articles in PubMed (up to November 21, 2017). The data quality was assessed by reviewing the scientific paper cited by the database as containing the experimental data and manually verifying that the stated interactions were experimentally described in the cited paper. Papers were identified from miRTarBase (October 2015) that were cited as containing evidence for the direct interaction of human miR-21 (hsa-mir-21-5p) with its mRNA targets based on reporter assay data, the current “gold standard” for validating miRNA targets (Nicolas 2011). A total of 163 miRNA:target interactions for hsa-mir-21-5p (representing 79 unique miRNA:target pairs) were listed in miRTarBase as reporter assay-evidenced, with 115 papers referenced. As our aim was to find one piece of supporting evidence from the cited papers for each of the unique miRNA:target interactions, once experimental evidence was verified for a specific target then no other cited papers were investigated for that miRNA:target pair. Consequently, 73 out of the 115 papers were reviewed. Out of the 109 miRTarBase interactions that were investigated, 30 (28%) did not have sufficient supporting evidence for a direct interaction (Table 1; Supplemental Table S1). The high number of interactions with weak or nonexistent evidence was surprising, given that these were categorized as reporter assay-evidenced interactions. Furthermore, while undertaking this investigation, an additional 29 unique gene targets of hsa-miR-21-5p were identified in the literature that were not included in miRTarBase. These reporter assay-evidenced interactions were reported in seven papers. Four of the seven papers were listed in miRTarBase as containing miR-21 target interactions, however not all of the targets were registered in miRTarBase. At the time of this investigation, miRTarBase cited papers with a publication date up to 2015, however another paper (Liu et al. 2012) with hsa-miR-21-5p reporter assay-evidenced data was not included at all in miRTarBase. This suggests that the pipeline used to populate this resource is missing valuable data, and the database has not captured all pre-2015 data. The miRTarBase curation procedure uses natural language processing as an initial screen to find the co-occurrence in a paper of miRNA and gene names, followed by manual review to confirm the suitability of the evidence (Chou et al. 2015). Nevertheless, there are some cases where this process has failed to find the appropriate experimental evidence. One example of this comes from the miRTarBase entry for the interaction between hsa-miR-221-3p and TMED7 (synonym “p27”), which cited nine papers as containing experimental evidence for this interaction. However, eight of these papers describe the interaction of miR-221 with the cyclin-dependent kinase inhibitor CDKN1B, which also has the synonym “p27.” The remaining paper provides evidence for SIRT1 as a target of miR-34a, but no evidence for the miR-221:TMED7 interaction. Therefore, there is no reliable cited experimental support for the interaction of miR-221-3p with TMED7 in miRTarBase, at the time of investigation.

**TABLE 1.**
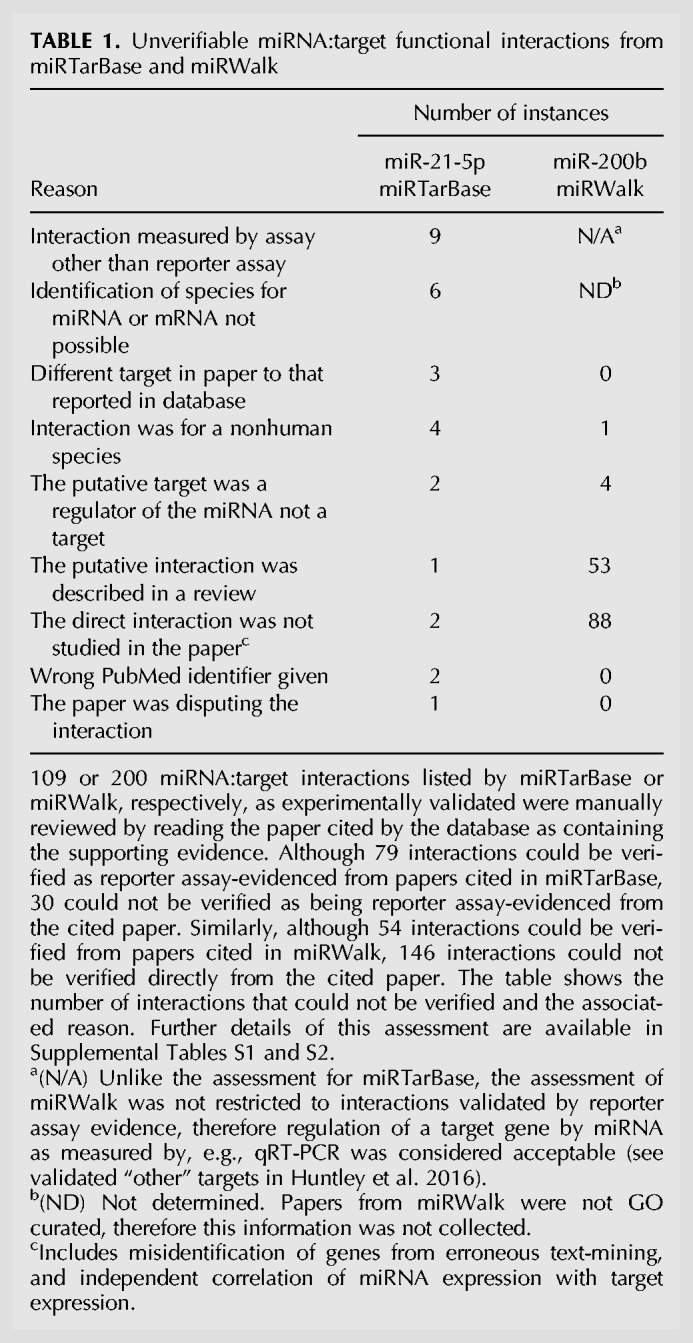
Unverifiable miRNA:target functional interactions from miRTarBase and miRWalk

A similar assessment was performed for experimentally validated targets of miR-200b cited in the miRWalk database, with equally concerning outcomes (Table 1; Supplemental Table S2). In July 2014, miRWalk reported 200 experimentally validated interactions (not restricted only to reporter assay evidence) for miR-200b, comprising 130 unique targets. After tracing the evidence for these interactions to the cited papers, only 54 interactions (27%), representing just 18 unique targets, could be verified as having experimental evidence support. The evidence for 53 of the 146 remaining interactions was derived from three review papers, not the original experimental evidence. These interactions may be correct, but it is impossible to confirm this without tracing back to the original data, thus, potentially up to 54% of the interactions reported in miRWalk for miR-200b are correct. The miRWalk database uses automated text-mining algorithms to identify miRNA:target interactions by surveying the title and abstract of papers in PubMed for co-occurrence of miRNA and gene names (Dweep and Gretz 2015). Although this is a useful first step in identifying potential miRNA targets, ideally there should be a manual verification step to confirm the association. An exclusively text-mining approach has previously been shown to have a >20% error rate in the case of finding protein–protein interactions from publications (Krallinger et al. 2008).

As a result of our manual verification, it is clear that two of the most popular miRNA target databases available to researchers contained a substantial degree of inaccurate data, at the time of investigation, that has the potential to mislead those wishing to find experimentally validated targets. Of course, for all data resources it is important for users to report any erroneous data directly to the resource to enable them to correct the errors. During our curation process we report the errors we find, which we hope helps to improve the data quality in the resource. Since this investigation, both miRTarBase and miRWalk have undergone major updates, therefore we are hopeful that some of the examples cited here have been corrected.

For scientists to be able to propose meaningful and evidence-supported hypotheses, it is necessary for them to access robust information. With high-quality, carefully curated functional data, such as we describe herein, more accurate investigation of miRNA roles in health and disease will be possible.

### The miRNA functional annotation resource

To begin to address the variable quality and paucity of miRNA functional information currently available to researchers, our project (the British Heart Foundation-funded functional gene annotation initiative at University College London: “BHF-UCL”) has created two novel bioinformatic data sets that provide reliable, experimentally verified functional information for mammalian miRNAs. Although our primary focus is curating the role of miRNAs involved in cardiovascular-related processes, many of the curated miRNAs have roles in diverse developmental, metabolic and signaling processes since they can each potentially target many hundreds of genes. Therefore, the biological roles we capture are relevant to many biological areas, including early development, cancer, as well as cardiovascular disease. Both data sets are curated from primary experimental literature by expert biocurators: The first data set consists of GO annotations assigned directly to miRNAs and the second is a molecular interaction data set of miRNAs and their validated target genes. Both data sets are described in more detail below. Annotation statistics for the resource are shown in Table 2; to date, we have created over 4400 GO term annotations for over 500 miRNAs from human, mouse and rat, through manual annotation of published experimental data. For human alone this provides over 3200 GO annotations for 372 miRNAs. Additionally, over 2400 experimentally validated miRNA:target interactions are available for use in network analysis. To illustrate the quality of the annotations that are provided by this resource, statistics for some of the GO term branches we have most frequently annotated to miRNAs are listed in Table 2 together with the evidence codes used (Balakrishnan et al. 2013). In order to provide only high-quality validated miRNA target information, the target gene is always identified in the annotation extension field of annotations that describe the interaction of a miRNA with its target gene (GO:0035195 and GO:1903231, see Table 3) and the targets are never inferred by sequence similarity (ISS) using data from another species. Biological processes and molecular functions that are regulated by the miRNA may be evidenced with ISS using strict criteria (described in “MiRNA functional annotation” section). In addition, the “Inferred from High-throughput Direct Assay” (HDA) evidence code is currently only used for carefully assessed, high-quality proteomics experiments describing evidence for cellular component annotations.

**TABLE 2.**
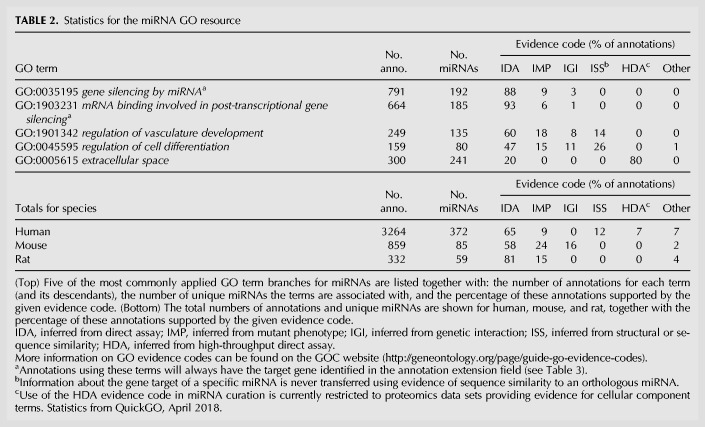
Statistics for the miRNA GO resource

**TABLE 3.**
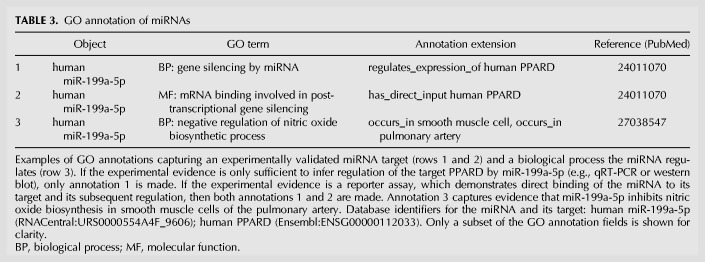
GO annotation of miRNAs

Our GO data set is now available via established database providers (e.g., miRBase, Ensembl, NCBI, GO Consortium) and, together with the molecular interaction data set (available via the PSICQUIC web service), can be incorporated into network analysis tools such as Cytoscape (see “Data set availability” section for information about access to each data set). Manual curation from primary literature is thorough, therefore time-consuming, but the benefit to researchers is that it obviates the need for them to source the original papers for confirmation of evidence. The biocurators are ideally placed to identify any discrepancies or errors in papers, which may raise concerns with the validity of the results, and report these problems to the relevant source. The biocurators use their expertise and judgment to determine whether the information is suitable for inclusion in the GO database, thereby reassuring researchers and bioinformaticians that the data provided in our resource is of the highest quality.

### MiRNA functional annotation

Our biocuration approach captures several aspects of miRNA function: their experimentally validated mRNA targets and the processes they regulate or are part of, combined with relevant contextual information where evidence is available. Here we describe how this information is curated and the value that this data adds to functional analysis of miRNAs.

#### Curation of experimentally validated miRNA targets

Identification of the “real” targets of a particular miRNA is frustrating, given the inconsistencies and inaccuracies of current miRNA target databases (Lee et al. 2015b). By utilizing only primary, published experimental data, we endeavor to provide freely accessible data consisting of high-quality, manually curated information about experimentally validated miRNA:target interactions. The process of capturing the target of a miRNA as a GO “annotation” has been described previously (Huntley et al. 2016; briefly described in Materials and Methods). An example of how experimental evidence supporting a miRNA:target interaction is represented as GO annotation is shown in rows 1 and 2 of Table 3.

**FIGURE 1.**
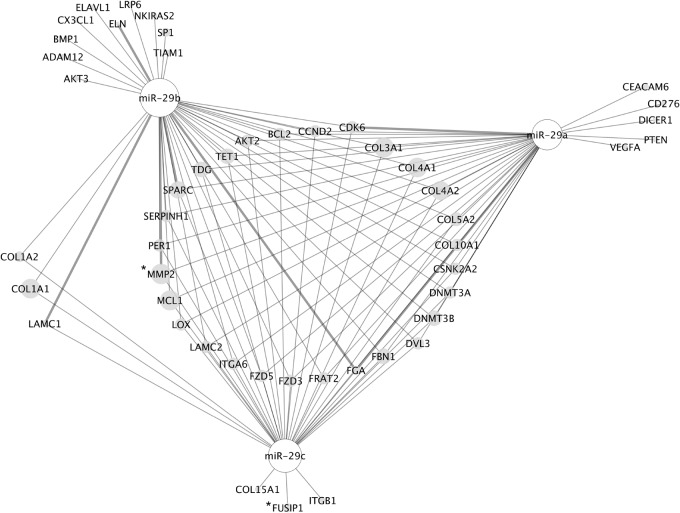
Experimentally verified targets of the miR-29 family. The network was created in Cytoscape (Shannon et al. 2003) using the miRNA:target association data set, “EBI-GOA-miRNA,” available from the PSICQUIC web service. All experimentally validated targets of the three miRNA-29s reported in the literature were curated prior to creating the network. The thin gray edges indicate direct interactions (interaction type = “physical association”); thick gray edges indicate where the directness of the interaction is unknown (interaction type = “association”). The gray nodes are gene targets regulated by the miRNAs; the white nodes are the miRNAs. The RNAcentral identifiers of the miRNAs are as follows: hsa-miR-29a-3p: URS00002F4D78_9606; hsa-miR-29b-3p: URS000024463E_9606; hsa-miR-29c-3p: URS0000272A3D_9606. (*) denotes proteins mentioned in the text.

To ensure maximum utility of these annotations, the miRNA:target interaction information in the GO annotation database was extracted to provide a PSICQUIC web service-compatible data set (“EBI-GOA-miRNA”) for use in network analysis tools such as Cytoscape (Shannon et al. 2003; del-Toro et al. 2013). While this data does not meet strict IMEx standards (Orchard et al. 2012), the data is manually curated from the primary experimental literature, therefore has more robust support than interaction data generated by text-mining. Our interaction data set, currently consisting of 2400 miRNA:target interactions, may be used to create networks of miRNAs and their experimentally validated targets. For example, an interaction network of the three members of the miR-29 family can be created (Fig. 1). By viewing the miRNA target data in this manner we may easily visualize useful information, such as the high number of confirmed mRNA targets of all three miRNAs (e.g., MMP2, indicated by [*] in Fig. 1) and those that are targeted by only one member of the miR-29 family (e.g., based on current knowledge, FUSIP1 is targeted only by hsa-miR-29c-3p, indicated by [*] in Fig. 1).

#### Curation of miRNA-regulated pathways and processes

One of the major uses of GO annotation data is in GO term enrichment analysis, which allows researchers to find the biological attributes that a set of gene products have in common. To make these analyses possible requires a substantial body of GO annotations describing the processes and functions that the miRNA is involved in. We take a process-focused approach to describe miRNA roles and functions in order to provide data that is maximally useful to the scientific community; areas curated so far include angiogenesis, early heart development and aneurysm-related processes. The biological processes and molecular functions that a miRNA is part of or involved in regulating are captured from the experimental literature as described previously (Huntley et al. 2016). An example of a typical GO annotation for a biological process regulated by a miRNA, including contextual details describing the cell and tissue in which the process is being regulated, is shown in row 3 of Table 3. Priority is given to biological processes that will be most beneficial to therapeutic applications, and within the process-based approach, priority is given to miRNAs that are proposed to be therapeutic targets and which have been selected for clinical trial, e.g., miR-15, the inhibition of which was shown to protect against cardiac ischaemia damage (Hullinger et al. 2012); miR-29, which is under investigation for stabilizing atherosclerotic plaques (Ulrich et al. 2016), alleviating pulmonary fibrosis (Montgomery et al. 2014) and providing a therapy for Duchenne muscular dystrophy (Heller et al. 2017); miR-155, which is being investigated for treating T cell lymphoma (Seto et al. 2015); and miR-208 under investigation for treatment of heart failure (Montgomery et al. 2011). Occasionally, experimental information is lacking for a given human miRNA, therefore experimental evidence from a mammalian ortholog may be curated instead. However, to prevent any unintended over-interpretation of the experimental outcomes we curate, GO annotations are not routinely transferred between miRNA family members, or to the orthologous miRNA in another species. Annotations are transferred to a second miRNA only if specific criteria are met which support the assertion that the function, or role, is likely to be conserved between the two miRNAs. This requires evidence of the following: (i) identical seed sequences; (ii) the miRNAs are predicted to target the same gene(s); (iii) the function or role is relevant for the species in which the orthologous miRNA exists. The resulting annotations, created by copying the annotations from one miRNA to the orthologous miRNA, will have the GO evidence code “Inferred from Sequence or Structural Similarity” (ISS) (Balakrishnan et al. 2013). Automatic application of GO annotation to miRNAs, e.g., by pipelines such as Ensembl Compara (Vilella et al. 2009), may be possible but we recommend careful consideration of whether the resulting functional statements are likely to be correct.

### The impact of miRNA functional annotation

**FIGURE 2.**
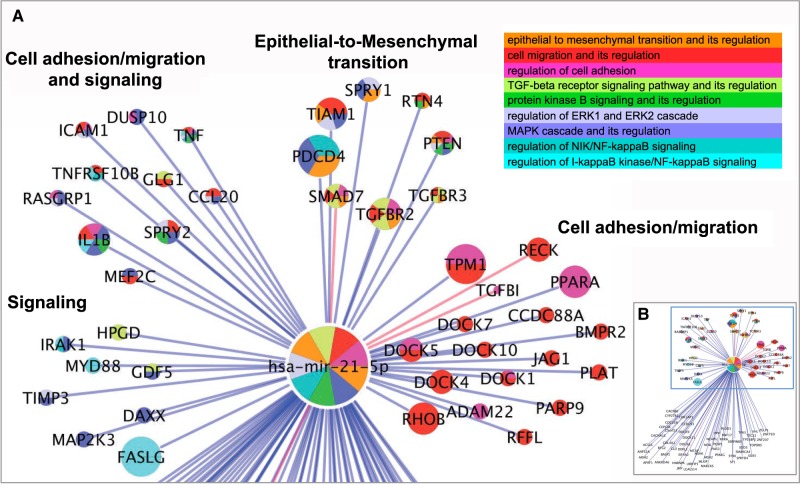
The role of miR-21 in epithelial-to-mesenchymal transition. (*A*) Part of the interaction network of hsa-miR-21-5p relevant to epithelial-to-mesenchymal transition (EMT). The network was created using Cytoscape (Shannon et al. 2003) and enriched GO terms identified using the plugins, GOlorize and BinGO (Supplemental Table S3; Maere et al. 2005; Garcia et al. 2007). Enriched GO terms relevant to EMT were selected and the miR-21 targets annotated to these terms or their descendants were clustered together according to the related processes they are involved in. Each entity (node) is color-coded according to the term(s) it is annotated to. Size of the nodes represents the number of times the interaction has been captured as an annotation. The blue edges indicate interaction type “physical association,” applied when the miRNA is demonstrated to bind the mRNA; red edges indicate interaction type “association,” applied when the experimental data does not demonstrate direct miRNA:target binding. (*B*) The full interaction network of hsa-miR-21-5p; the boxed area shows the target interactions relevant to EMT, as shown in *A*.

To demonstrate how the annotations in our two data sets have impacted analysis of miRNA function, hsa-miR-21-5p was used as an example. Mir-21 has been very well studied and has been shown to be crucial in many biological pathways and diseases, including cardiovascular disease and several cancers (Kumarswamy et al. 2011; Pfeffer et al. 2015). MiR-21 is transcribed from the MIR21 gene and gives rise to two mature miRNAs, miR-21-3p and miR-21-5p. MiR-21-5p is the most abundant transcript and consequently, the best studied (http://mirbase.org/cgi-bin/mirna_entry.pl?acc=MI0000077). So far, 98 experimentally validated targets of hsa-miR-21-5p have been identified from the available experimental literature and curated into our GO annotation and miRNA:target interaction data sets (Fig. 2B). With such a high number of targets, it is not surprising that miR-21 is involved in regulating many different processes and pathways, however, this makes it challenging to visualize all of miR-21's regulatory roles simultaneously. A more informative way to look at the data is to focus on a process or pathway of interest, which effectively narrows down the number of targets, therefore gaining insight into the functional role of a miRNA and its targets in the specified process. This approach was taken to visualize the role of hsa-miR-21-5p in the epithelial-to-mesenchymal transition (EMT).

#### Visualizing the role of miR-21 in epithelial-to-mesenchymal transition

Several miRNAs are known to regulate the EMT, a process where epithelial cells lose their cell adhesion properties, gain migratory properties and become mesenchymal cells; multipotent stem cells which can differentiate into a variety of cell types (Kalluri and Weinberg 2009; Zhao et al. 2017). EMT is important for the development of many organs, including the heart, and is also involved in fibrosis and in the development of cancer (Brønnum et al. 2013; Li et al. 2016; Zou et al. 2017). EMT is regulated by many signaling pathways including nuclear factor kappa-B (NF-κB), mitogen-activated protein kinase (MAPK), phosphatidylinositol 3-kinase/protein kinase B (PI3K/PKB) and transforming growth factor beta/mothers against decapentaplegic (TGFβ/SMAD) (Li et al. 2016; Brønnum et al. 2013) and emerging evidence suggests that miR-21 has pleiotropic roles in regulating these pathways, in addition to regulating EMT itself, through the regulation of its many gene targets. For example, in cardiac fibroblasts miR-21 silencing of PTEN results in PKB phosphorylation and the subsequent activation of MMP2, a known inducer of EMT (Gilles et al. 2000; Roy et al. 2009; Radisky and Radisky 2010). This corroborates an earlier finding that PTEN inhibits EMT through PI3K and PKB signaling (Wang et al. 2007). Furthermore, miR-21 silencing of its targets SPRY1, PDCD4 and SMAD7, was shown to partly contribute to fibrogenic EMT induced by TGFβ (Brønnum et al. 2013; Wang et al. 2014). TGFβ itself promotes the processing of primary mir-21 to precursor miR-21 via a SMAD-dependent signaling pathway (Davis et al. 2008).

Thus, the role of miR-21 in regulating EMT is complex, however, by curating the primary experimental evidence describing these processes, the resulting data set of GO annotations associated directly with miR-21 and its targets can be used to begin to obtain a clearer picture of how miR-21 contributes to regulating EMT. In order to visualize the role of miR-21 in EMT, it was first necessary to create the interaction network of miR-21-5p with all of its validated gene targets using Cytoscape (Shannon et al. 2003). Following this, the Cytoscape plugins, BinGO, and GOlorize (Maere et al. 2005; Garcia et al. 2007), were used to overlay a GO term enrichment analysis onto the network and highlight the biological processes that are involved in EMT. Figure 2A shows the part of the interaction network that contains targets of hsa-miR-21-5p that are involved in processes relevant to EMT, such as “epithelial to mesenchymal transition,” “cell adhesion” and “cell migration,” as well as various signaling pathways, which were identified as enriched in this network (the full interaction network of hsa-miR-21-5p is shown in Fig. 2B and the full BinGO enrichment results are in Supplemental Table S3). In addition to providing an improved view of the participants in EMT and its regulation, this network can assist both with identifying missing information and with inferring putative roles for individual proteins. As an example of information missing from the GO database, interleukin-1 beta (IL1B) is annotated to regulation of NF-κB and MAPK signaling as well as cell migration and regulation of cell adhesion, all indicative of involvement in EMT, however IL1B was not represented in GO as being involved in EMT. A search of the literature quickly found evidence of this role for IL1B; expression of the EMT markers SNAI1, SNAI2, and VIM is induced by IL1B in oral squamous cell carcinoma cells and increases migration of these cells (Lee et al. 2015a). This information has now been added to the GO database, thereby making it easily accessible for future analyses.

Putative roles of individual proteins in regulating the reprogramming of epithelial cells during EMT may also be inferred from their existing GO annotations. An example from Figure 2A is the dual specificity protein phosphatase 10 (DUSP10), which is well characterized as an inactivator of MAP kinases and the MAPK signaling pathway (Zhang et al. 2011) and also involved in regulatory T cell differentiation (Chang et al. 2012); both of these roles are already represented with GO annotation. However, DUSP10 has also been shown to inhibit cell migration in various cells (Song et al. 2013; Png et al. 2016), and as an increase in migration is one of the key indicators of EMT, it is possible that DUSP10 is involved in regulating EMT. The role of DUSP10 in regulating migration was not represented in GO at the time of our analysis, consequently we have ensured it is now available. Together, these roles suggest that DUSP10 may have a previously unrecognized role in EMT, which is not yet reported in the experimental literature, although other DUSP family members have been associated with EMT (Boulding et al. 2016).

## DISCUSSION

There are numerous resources providing various types of data for miRNAs, most of which include a combination of predicted and experimental evidence concerning sequence, expression and targets (for a review, see Akhtar et al. 2016), but there are few that provide reliable, experimentally based functional data for miRNAs that is both human- and computer-readable. This article demonstrates the need for high-quality functional annotation for miRNAs by presenting examples of inconsistencies that are found in existing miRNA target databases, largely due to the lack of a direct link between the experimental literature and the information in the miRNA database. One resource that can provide this direct link is GO, which has already proven essential for navigating the knowledge of protein-coding genes (a search of PubMed with the phrase “gene ontology” identified >11,400 papers in April 2017); our data set extends this collection of gene products to include miRNAs. One of the major uses of GO annotation data is in GO term enrichment analysis, which supports the identification of commonalities in a list of gene products. For example, a researcher may wish to determine the roles of a list of miRNAs that are differentially regulated in a specific disease in order to discover which processes or pathways are affected. Prior to the creation of our miRNA GO annotation data set, it was impossible to perform standard GO term enrichment analyses on the verified roles of miRNAs, due to the lack of computationally accessible functional information. Instead, researchers carry out the analysis on the predicted targets of the miRNAs, which can number into the thousands. Numerous studies of this kind have been published (for a selection, see Bleazard et al. 2015), however it has been demonstrated that the most common approach currently used for miRNA pathway analysis is biased toward cell cycle and cancer pathways, regardless of the condition or disease of interest (Godard and van Eyll 2015).

At present, all human miRNA GO annotations available through miRBase (Kozomara and Griffiths-Jones 2014), Ensembl (Yates et al. 2016), NCBI Gene (Brown et al. 2015), the GO Consortium (The Gene Ontology Consortium 2017) and miRNA:target interactions available from the PSICQUIC web service (del-Toro et al. 2013) have been created by the BHF-UCL functional gene annotation initiative. With our data, users can be confident that the interactors shown have been experimentally validated and are not based on computational prediction or text-mining. Furthermore, with the increasing number of experimentally validated functional annotations that our project associates directly with miRNAs, more meaningful enrichment analyses of miRNAs is within sight. The GO annotations can be incorporated into networks, created with our curated miRNA:target interactions, to determine which processes or pathways the miRNAs directly regulate through these interactions.

Using the miRNA functional annotations that are freely available in our resource, we have illustrated the positive impact that these can have on functional and network analyses. The curation approach we use can be used by anyone wishing to improve representation of miRNA function in any area of biology for any species. Our miRNA resource is at an early stage, but as it expands, it will further increase the visibility of the miRNA-focused experimental research being published, allowing it to be included in the most commonly used analysis tools, such as DAVID and g:Profiler. MiRNA functional analysis will therefore become increasingly meaningful and accurate, thus informing hypotheses for future research into disease therapies.

### Future work

Functional analysis of gene products requires a substantial body of annotation to provide statistically significant results, therefore ongoing biocuration of miRNAs will continue to provide additional annotations, ensuring well-populated and high-quality data sets that complement the existing GO and molecular interaction resources for genes and proteins. MiRNAs are increasingly being studied for their therapeutic potential in cardiovascular, and many other, diseases, so it is critical that the results of these studies are reflected in bioinformatic resources. To date, miRNAs involved in angiogenesis, early heart development and aneurysm-related processes have been curated. In order for our resource to be of maximum utility to translational medicine and enhance the efforts for providing therapeutics targets, our biocuration will continue to focus on pathways and processes that are targets for therapeutic applications.

Maximizing the use of our miRNA GO annotations is one of our key objectives. Consequently, we are in discussions with several high-profile bioinformatic resource providers to enable the inclusion of the annotations in their databases or analysis tools, including RNAcentral (The RNAcentral Consortium 2015), DAVID (Huang et al. 2008), g:Profiler (Reimand et al. 2016) and PANTHER (Mi et al. 2013). Since many functional analysis tools obtain their GO annotation data from Ensembl (e.g., g:Profiler), NCBI (e.g., DAVID), or directly from the GO Consortium annotation files (e.g., PANTHER, VLAD [Richardson and Bult 2015], Ontologizer [Bauer et al. 2008]), we anticipate that modification of these tools to incorporate miRNA GO annotations will require relatively low investment by the providers. Combining miRNA, gene, protein and macromolecular complex annotations within the same analysis tools will enable more complex data sets to be analyzed, for example, noncoding and coding transcriptomic data.

Finally, community biocuration has proven extremely successful for certain biological communities, e.g., *Schizosaccharomyces pombe* (Rutherford et al. 2014) and *Arabidopsis thaliana* (Berardini et al. 2012); we hope to leverage the collaborative spirit of the miRNA community in order to engage researchers with improving bioinformatic resources through biocuration. One way forward with this is the GO curation of experimentally validated miRNA:target interactions, which follows a strict set of guidelines that can be easily quality checked. Development of a simple tool for use by researchers and authors to capture published experimentally validated miRNA targets will be investigated for this purpose. Researchers wishing to contribute to these resources now can send primary research papers suitable for biocuration to us at goannotation@ucl.ac.uk.

## MATERIALS AND METHODS

### Curation procedure

In order to create the data set of miRNA functional annotation using GO vocabulary, standard GO annotation procedures were followed, in addition to adhering to the guidelines for biocuration of the functional roles of miRNAs and their experimentally validated target genes (Balakrishnan et al. 2013; Huntley et al. 2014, 2016) (http://wiki.geneontology.org/index.php/MicroRNA_GO_annotation_manual). One of the most important criteria for curating a miRNA is the identification of the miRNA sequence used in experimental assays from any given paper, which is used by biocurators to find the appropriate identifier in RNAcentral (The RNAcentral Consortium 2015) to associate GO terms with. The seed sequence is a major contributor for miRNA interaction with 3′ UTR target gene sequences; an alteration of just one nucleotide of the seed sequence can change the spectrum of the mRNAs targeted by the miRNA by over 50% (Hughes et al. 2011; Hill et al. 2014). This difference in target spectrum can lead to either regulation of alternative processes than is usual for the miRNA, and/or differential regulation of a process or pathway the miRNA usually regulates. If the sequence of the miRNA is not reported in the paper, or it is not traceable through a citation or a product catalog number, then the experimental data relating to that miRNA cannot be curated. It is, therefore, essential that authors provide an exact sequence for all miRNAs studied, so that their experimental data can be correctly represented in bioinformatic databases.

### Curation approach

To maximize the value of the annotations to the research community, a biological process-based approach is taken to curating miRNAs (Alam-Faruque et al. 2011). This approach involves taking a specific process or pathway and curating all miRNAs that have been experimentally demonstrated as having a role. This allows the functions and roles of many miRNAs to be covered in the context of that process or pathway and provides a comprehensive representation of that knowledge in the GO database. Within this approach, published papers that include experimentally verified functional data are prioritized for curation. On occasion a miRNA-centric approach is taken, which provides detailed knowledge about a single miRNA and its involvement in a variety of processes. The miRNA-centric approach is time-consuming—each miRNA can target hundreds of miRNAs and therefore affect many processes—but does not provide a complete insight into any single process; therefore the process-centric approach is regarded as providing the most impactful information.

### Molecular interaction data set for miRNAs and their targets

A molecular interaction data set (“EBI-GOA-miRNA”) was created in PSI-MI format and made available on the PSICQUIC web service, to enable computational access to miRNA interactions with their experimentally validated targets. The source of the information in this data set is GO annotations that we have created containing experimentally verified miRNA:target interaction data. GO annotations used for this purpose conform to the following criteria; the “Database Object ID” field of the GO annotation file must be a miRNA, specified by an RNAcentral ID, AND the “Annotation Extension” field must contain an mRNA target, specified by an Ensembl gene ID (del-Toro et al. 2013; Huntley et al. 2014). For the PSICQUIC specification, those interactions described with the Molecular Function GO term *mRNA binding involved in post-transcriptional gene silencing* (GO:1903231) are assigned interaction type “physical association,” indicating direct binding of the miRNA to the mRNA target. Interactions described only with one of the following GO Biological Process terms: *gene silencing by miRNA* (GO:0035195); *miRNA mediated inhibition of translation* (GO:0035278); *mRNA cleavage involved in gene silencing by miRNA* (GO:0035279); *deadenylation involved in gene silencing by miRNA* (GO:0098806), but without the Molecular Function term above are assigned the interaction type “association,” indicating that the evidence demonstrated miRNA regulation of the target only (see “Data set availability” section below for the file format information and access to this data set).

### Network analyses

Details of the data sets and software used are given to allow reproduction of these analyses.

#### Interaction network of miR-29 family

The molecular interaction network of the miR-29 family was created in Cytoscape v3.2.1 using our “EBI-GOA-miRNA” molecular interaction data set (January 2017). The interaction network was seeded with the three miR-29 family identifiers from RNAcentral: hsa-miR-29a-3p: URS00002F4D78_9606; hsa-miR-29b-3p: URS000024463E_9606; hsa-miR-29c-3p: URS0000272A3D_9606.

#### Functional analysis of miR-21 and its targets

The molecular interaction network of hsa-miR-21-5p (URS000039ED8D_9606) was created in Cytoscape using our “EBI-GOA-miRNA” data set (March 2017). GO term enrichment was subsequently performed on the network using the Cytoscape plugins BinGO (Maere et al. 2005) and GOlorize (Garcia et al. 2007). The files used in the GO enrichment were as follows: Gene Ontology; go-basic.obo (May 10, 2017) downloaded from the GO Consortium website (http://geneontology.org/page/download-ontology), the gene association files goa_human_rna.gaf and goa_human.gaf from May 8, 2017 were downloaded from the EMBL-EBI ftp site (ftp://ftp.ebi.ac.uk/pub/databases/GO/goa/HUMAN/) and merged into a single file before upload into the BinGO application.

## DATA DEPOSITION

The molecular interaction data set, “EBI-GOA-miRNA,” is available from the PSICQUIC web service (http://www.ebi.ac.uk/Tools/webservices/psicquic/view/home.xhtml) and the QuickGO web service (http://www.ebi.ac.uk/QuickGO/psicquic-rna/webservices/current/search/interactor/*) or from directly within Cytoscape. The data set is in PSI-MITAB 2.7 format, which is described at https://psicquic.github.io/MITAB27Format.html. MiRNA GO annotations are deposited in the UniProt-GOA database via the curation tool Protein2GO (Huntley et al. 2015) using RNAcentral identifiers to indicate the species-specific miRNA, e.g., RNAcentral:URS000039ED8D_9606 identifies human miR-21-5p. The annotations are distributed in Gene Association Format 2.1 (GAF2.1) and Gene Product Association Data format 1.1 (GPAD1.1) annotation files, which can be downloaded from the UniProt-GOA ftp site (ftp://ftp.ebi.ac.uk/pub/databases/GO/goa/) from the relevant species file; e.g., the annotations for human miRNAs are found in the files goa_human_rna.gaf and goa_human_rna.gpa for GAF2.1 and GPAD1.1 format, respectively. These files are updated every four weeks at which time the GAF2.1 file is also distributed to the GO Consortium ftp site (ftp://ftp.geneontology.org/pub/go/gene-associations/). The project is funded by the British Heart Foundation (BHF) to create cardiovascular-related GO annotations; therefore, these annotations can be identified by the source “BHF-UCL” located in the “Assigned_By” field of the GAF2.1 and GPAD1.1 files. The miRNA annotations can also be accessed via the UniProt GO browser QuickGO and the GO Consortium's AmiGO browser; e.g., the entry for hsa-miR-21-5p can be viewed in QuickGO at http://www.ebi.ac.uk/QuickGO/annotations?geneProductId=URS000039ED8D_9606 and in AmiGO at http://amigo.geneontology.org/amigo/gene_product/RNAcentral:URS000039ED8D_9606.

## SUPPLEMENTAL MATERIAL

Supplemental material is available for this article.

## Supplementary Material

Supplemental Material
