# Supplementary material for: Expanding the horizons of microRNA bioinformatics
Source: RNA. 2018 Aug;24(8):1005–17. doi: 10.1261/rna.065565.118 (PMC6049505; doi:10.1261/rna.065565.118)
Supplement: Supplemental Material [file supp_24_8_1005__index.html]

Expanding the horizons of microRNA bioinformatics — Supplemental Material 

# Expanding the horizons of microRNA bioinformatics

## Supplemental Material

- Supplemental\_Table\_Legends.docx
- Supplemental\_Table\_S1.xlsx
- Supplemental\_Table\_S2.xlsx
- Supplemental\_Table\_S3.xlsx
