# Supplementary material for: Expanding the horizons of microRNA bioinformatics
Source: RNA. 2018 Aug;24(8):1005–17. doi: 10.1261/rna.065565.118 (PMC6049505; doi:10.1261/rna.065565.118)
Supplement: Supplemental Material [file supp_065565.118_Supplemental_Table_Legends.docx]

**SUPPLEMENTARY MATERIAL**

Supplementary Material is available online:

Supplemental_Table_S1. *Assessment of validated gene targets for miR-21-5p as reported in miRTarBase.* Papers cited by miRTarBase (October 2015) as containing direct, reporter assay-evidenced interactions of human miR-21-5p with putative mRNA targets were identified and manually checked by a curator for the cited interaction. The data presented in the table is the downloaded information from miRTarBase, appended with the curators’ assessment of the validity of the reported miRNA:mRNA interaction.

Supplemental_Table_S2. *Assessment of validated gene targets for miR-200b as reported in miRWalk.* Papers cited by miRWalk (July 2014) as containing experimentally evidenced, direct interactions of human miR-200b with putative mRNA targets were identified and manually checked by a curator for the cited interaction. The data presented in the table is the downloaded information from miRWalk, appended with the curators’ assessment of the validity of the reported miRNA:mRNA interaction.

Supplemental_Table_S3. *BinGO term enrichment for epithelial-to-mesenchymal transition.* Includes the full enrichment results of the BinGO analysis displayed in Figure 2. GO terms that were enriched based on 2 or fewer gene products have been removed.
